# Barriers and facilitators of adherence to cancer prevention in Lynch Syndrome: an integrative review

**DOI:** 10.1186/s13053-026-00348-9

**Published:** 2026-07-17

**Authors:** Marjorie Matekwa, Margaret H. White, Janet H. Van Cleave, Veronica J. Brady, Carina Katigbak

**Affiliations:** Cizik School of Nursing, The University of Texas Health Science Center at Houston (UTHealth Houston), Houston, TX USA

**Keywords:** Lynch Syndrome, Cancer prevention, Early detection of cancer, Adherence, Barriers, Facilitators, Primary care

## Abstract

**Background:**

Lynch Syndrome, the most common hereditary cancer syndrome affecting 1 in 279 Americans, relies on primary care providers to identify at-risk individuals and promote adherence to prevention strategies. However, adherence remains suboptimal. This review examines barriers and facilitators impacting adherence for individuals with Lynch Syndrome and their primary care providers.

**Methods:**

A systematic search of six electronic databases (Embase, PubMed, MEDLINE, Google Scholar, CINAHL, and Web of Science) was conducted for studies published between 2011 and 2024. Studies examining barriers or facilitators to cancer prevention among individuals with Lynch Syndrome or primary care providers were included. Methodological quality was assessed using the Joanna Briggs Institute criteria for qualitative studies.

**Results:**

Four studies met the inclusion criteria (sample sizes 13–60). Key barriers included limited provider and patient knowledge of Lynch Syndrome, psychological distress related to cancer risk, uncertainty surrounding follow-up protocols, and competing lifestyle priorities. Facilitators included strong wellness motivation, family support, and coordinated care pathways that support surveillance and preventive care.

**Conclusions:**

Adherence to cancer prevention in Lynch Syndrome is influenced by psychosocial factors and provider knowledge gaps. Addressing these barriers through targeted education and coordinated care models may improve adherence to prevention in this high-risk population.

## Introduction

Lynch Syndrome represents the most prevalent hereditary cancer syndrome, affecting approximately 1 in every 279 individuals across the United States [1]. This autosomal dominant condition arises from mutations in mismatch repair genes MLH1, MSH2, MSH6, PMS2, and EPCAM, predisposing carriers to a spectrum of up to 12 different cancers [2, 3]. Of all hereditary colorectal cancers, about 80% are associated with a Lynch Syndrome mismatch repair [4].

Recent prospective studies have documented substantial cancer risks among individuals with Lynch Syndrome. MLH1 mutation carriers face colorectal cancer risk that increases from 12.9% at age 40 to 46.7% at age 75 [5]. MSH2 carriers demonstrate higher endometrial cancer incidences, ranging from 1.5% at age 40 to 56.7% at age 75 [5]. The risk of upper gastrointestinal tract cancers, including stomach and duodenum malignancies, is also significantly elevated, particularly in the MSH2 and MLH1 pathways, respectively [5].

### Adherence to cancer prevention in Lynch Syndrome

Adherence to cancer prevention refers to the extent to which individuals with Lynch Syndrome follow recommended screening schedules, maintain preventive medications, implement lifestyle changes, and engage in risk-reducing behaviors as advised by healthcare providers [2, 6, 7]. Cancer prevention in Lynch Syndrome encompasses multiple evidence-based strategies, including regular surveillance through colonoscopy and endometrial screening, chemoprevention with aspirin therapy, prophylactic surgeries, and lifestyle modifications [8]. For primary care providers, adherence encompasses consistent implementation of Lynch Syndrome care guidelines, appropriate referrals to genetic counseling, coordination of multi-specialty care, and ongoing patient education and support [7].

Despite the availability of these strategies, adherence to recommended cancer prevention remains suboptimal among individuals with Lynch Syndrome. Studies report colonoscopy adherence rates ranging from 60% to80%, significantly below the ideal 100% target for this high-risk population [8]. Genetic counseling uptake varies from 34% to 52%, while screening adherence differs substantially by cancer type and healthcare setting [2, 6, 8].

Current prevention efforts have primarily focused on genetic counseling, vaccine development, adherence to screening, and surgical prophylaxis [8]. However, research addressing both individual and provider perspectives on cancer prevention barriers and facilitators remains limited. While some studies have focused on provider-specific challenges [7–9], a **notable gap** exists in comprehensive interventions that target specific behavior modifications to reduce the risk of cancer development among individuals with Lynch Syndrome, while simultaneously addressing provider implementation barriers.

These substantial prevention challenges underscore the critical importance of understanding and addressing the multi-level factors that influence adherence to cancer prevention strategies in Lynch Syndrome. Successful implementation requires a comprehensive understanding of the barriers and facilitators that influence adoption and sustained engagement with prevention behaviors among both carriers and their healthcare providers.

This integrative review aims to: (1) identify barriers and facilitators to cancer prevention adherence among individuals with Lynch Syndrome, (2) examine provider-specific factors influencing prevention care delivery, and (3) provide evidence-based recommendations for developing effective behavior change interventions in this high-risk population.

### Theoretical framework

The Health Belief Model (HBM), developed by Irwin M. Rosenstock [10], guided the conceptual framework for this review and influenced both the selection of search terms and the organization of the findings. The HBM suggests that health behaviors are shaped by individuals’ perceptions of their disease risk and its seriousness, as well as by cues to action that encourage preventive behaviors. The model’s main components: individual perceptions, modifying factors, and likelihood of taking action, were adapted to explore barriers and facilitators to adhering to cancer prevention among individuals with Lynch Syndrome (Appendix A). In this framework, individual perceptions were reflected in the family history of Lynch Syndrome and confirmed diagnoses, indicating perceived susceptibility to cancer. Perceived seriousness related to the emotional toll of cancer experiences within families, while perceived threats involved awareness of cancer risk that could motivate prevention efforts or lead to avoidance. Cues to action included access to healthcare, awareness, social support, and guidance from primary care providers that facilitated engagement in prevention strategies.

Modifying factors included demographic characteristics (age, sex, race, ethnicity, and nationality), socio-psychological influences (education and social context), and structural factors (prior knowledge of Lynch Syndrome, family exposure to cancer, and healthcare experiences). The likelihood-of-action component reflected the balance between perceived benefits of preventive behaviors and barriers to implementation. These constructs informed the development of the search strategy and guided the synthesis of barriers and facilitators influencing cancer prevention behaviors among individuals with Lynch Syndrome and healthcare providers (Appendix A).

## Methods

### Literature search strategy

A comprehensive literature search was conducted to identify English-language articles that address barriers and facilitators to cancer prevention among adults with Lynch Syndrome and primary care providers, including studies from both within and outside the United States. The search strategy was designed to capture the full scope of relevant literature addressing prevention challenges and opportunities in this specialized population.

The literature search involved a systematic exploration of multiple databases, including Embase, PubMed, MEDLINE, Google Scholar, Cumulative Index to Nursing and Allied Health (CINAHL), and Web of Science. This multi-database approach ensured comprehensive coverage of medical, nursing, psychological, and health services research literature relevant to Lynch Syndrome cancer prevention.

Search terms were developed iteratively, guided by the modified HBM theoretical framework (Appendix A). They included combinations of primary concepts related to the condition, prevention behaviors, and study populations. The terms “Lynch Syndrome” and “hereditary cancer syndrome” were combined with descriptors such as “cancer,” “neoplasm,” and “malignancy.” Prevention and adherence concepts were represented using terms such as “adherence,” “prevention,” “facilitator,” and “barrier.” Population-specific terms focused on “individuals with Lynch Syndrome” and “primary care providers,” recognizing the importance of both patient and provider perspectives.

### Inclusion and exclusion criteria

Articles were included if they: (1) were published between 2011 and 2024, ensuring focus on contemporary prevention challenges while maintaining sufficient publication volume; (2) had abstracts and titles relevant to the listed search terms; (3) specifically addressed barriers and facilitators to cancer prevention among individuals with Lynch Syndrome; (4) included primary care provider perspectives; and (5) were written in English to facilitate interpretation and ensure relevance to United States healthcare contexts.

Articles were excluded if they: (1) were published before 2011; (2) lacked individuals with Lynch Syndrome in the study population; (3) included barriers and facilitators from stakeholders without capturing the exclusive perspectives of individuals with Lynch Syndrome or primary care providers; or (4) focused on barriers and facilitators from caregivers to individuals with Lynch Syndrome (parents, spouses, or other relatives) as these could not capture the actual individuals’ lived experiences and decision-making processes.

### Study selection process

The literature search yielded 18 articles that met the initial search terms (Appendix B). These articles were systematically uploaded into the Covidence software (Covidence, [11]) to facilitate a transparent and systematic review of titles and abstracts. During initial screening, 10 studies were excluded because they addressed barriers and facilitators not specifically relevant to individuals with Lynch Syndrome or did not focus on cancer prevention behaviors. Four additional studies were excluded due to inappropriate study populations (including Lynch Syndrome Stakeholders and caregivers) or inadequate study designs (focused on barriers and facilitators to cascade testing for Lynch Syndrome).

This systematic process resulted in four articles meeting the full inclusion criteria for comprehensive review and analysis. Quality assessment was conducted using the Joanna Briggs Institute (JBI) Critical Appraisal Checklist for Qualitative Research [12], which was appropriate because all included studies employed qualitative research designs (Appendix C). Data extraction was conducted by two independent reviewers to enhance reliability and minimize bias. MM served as the primary reviewer and conducted the initial data extraction, while MHW independently reviewed each study using a standardized coding sheet developed a priori (Appendix D). The coding sheet ensured consistent and systematic extraction of study information, including study characteristics, research design, theoretical framework, variables of interest, sample characteristics, data analysis methods, key outcomes, identified barriers and facilitators to cancer prevention, data interpretation, and clinical implications. Following independent extraction, the reviewers compared their findings and resolved any discrepancies through structured discussion until consensus was achieved on the final dataset. This standardized approach enhanced the consistency, transparency, and methodological rigor of the review process.

## Results

### Study and participant characteristics

Studies were conducted across multiple countries: two in the United Kingdom, one in the Netherlands, and one in the United States (Table 1). All employed qualitative research designs, with one using a nested qualitative design within a larger research program [13]. Three studies employed semi-structured interviews [1, 9, 13] while one included focus groups [2].

**Table 1:** Main findings

Citation	Country	Study Aim	Study Design / Methodology	Type of Analysis	Population / Sample	Key Barriers	Key Facilitators
Kohut et al. [13].	United Kingdom	Examine provider–patient shared decision-making for cancer prevention among Lynch Syndrome carriers, including aspirin therapy and prophylactic gynecologic surgery.	Qualitative study (Nested)	Thematic analysis	L.S carriers and PCPs	Limited resources; sustainability concerns; language and cultural barriers; limited understanding of prevention benefits; lack of implementation evidence	Trusted provider knowledge; improved decision support; increased patient understanding; reduced decision regret
Lloyd et al. [9].	United Kingdom	Explore barriers and facilitators to aspirin use for cancer prevention among Lynch Syndrome carriers and primary care providers.	Qualitative study	Thematic analysis (NVivo)	L.S carriers and PCPs	Concerns about aspirin-related harm; uncertainty regarding the benefits of high-dose aspirin; limited clinical evidence; unfamiliarity with aspirin prevention; reluctance to discuss aspirin with providers; need for additional support	National guideline recommendations (NICE); provider agreement on prescribing pathways; high patient knowledge; patient preference for aspirin prevention
Srinivasan et al. [1].	United States	Explore stakeholder perspectives on barriers to cascade testing for Lynch Syndrome among carriers and their relatives.	Qualitative study	Thematic analysis (Atlas.Ti)	L.S carriers, PCPs, geneticists, and advocacy organizations	Low awareness of Lynch Syndrome; psychological barriers (fear, information avoidance); reluctance to disclose diagnosis; limited provider follow-up; limited access to testing and counseling; fear of employment or insurance discrimination	Familial motivation for testing; availability of free genetic testing
Visser et al. [2].	Netherlands	Examine the barriers and facilitators to adherence to WCRF/AICR cancer-prevention lifestyle recommendations among carriers of Lynch Syndrome.	Qualitative study	Thematic analysis	L.S carriers	Desire to maintain quality of life; illness burden; gastrointestinal symptoms from prevention interventions; social influences promoting unhealthy behaviors; limited time for healthy lifestyles; perceived loss of control	Improved well-being from healthy lifestyles; healthier dietary behaviors; improved fitness and endurance; longevity motivation; increased cancer risk awareness; family role modeling

Note. PCP - Primary Care Providers; L.S - Lynch Syndrome; WCRF - World Cancer Research Fund; AICR - American Institute of Cancer Research; NICE - National Institute of Healthcare Excellence

Data analysis approaches demonstrated methodological rigor: two studies used thematic analysis in Atlas-Ti software, one employed NVivo, and another used the MOSCOW prioritization method. All studies incorporated theoretical frameworks grounded in human behavior change.

Sample sizes ranged from 13 to 60 participants, with ages spanning from 18 to 70 years. Individuals with Lynch Syndrome ranged from 13 to 23, while primary care providers ranged from unspecified numbers to 23 providers. Most participants were female and identified as White. The average time since Lynch Syndrome diagnosis was seven years among carriers.

Recruitment utilized multiple channels: purposive sampling via Lynch Syndrome UK [9], social media platforms [1], ongoing research studies including CanGene-CanVar [13] and GeoLynch [2] and email outreach to providers using snowball sampling.

### Barriers and facilitators analysis

#### Patient-level barriers

Patient-level barriers to cancer prevention among individuals with Lynch Syndrome included knowledge deficits, psychological challenges, and lifestyle constraints [1, 2, 9, 13]. Many individuals demonstrated limited understanding of genetic risk and prevention strategies, and the complexity of genetic information contributed to uncertainty about testing and prevention decisions [2, 9, 13]. Psychological concerns, including fear of discrimination, cancer-related anxiety, and information avoidance, influenced motivation to pursue genetic testing and adhere to prevention recommendations [1, 2]. Lifestyle and social factors, such as competing priorities and concerns about maintaining quality of life, further hindered sustained engagement in recommended prevention behaviors [2].

#### Patient-level facilitators

Motivational and social factors served as key facilitators of sustained engagement in cancer prevention among individuals with Lynch Syndrome [1, 2]. Wellness motivation, a desire for longevity, and increased awareness of cancer risk following diagnosis strengthened individuals’ intrinsic motivation to adopt preventive health behaviors [2]. Family and social support further reinforced prevention efforts, with many individuals motivated by the desire to reduce family worry, serve as positive role models, and respond proactively to a family history of cancer [1, 2].

#### Provider-level barriers

Healthcare providers faced significant barriers, including knowledge gaps, clinical uncertainty, and structural limitations, when delivering cancer prevention services to individuals with Lynch Syndrome [1, 9, 13]. Providers reported uncertainty regarding appropriate follow-up protocols and optimal prevention strategies, including aspirin use, screening intervals, and coordination of comprehensive care [9, 13]. System-level barriers further limited prevention efforts, including the cost of diagnostic and follow-up care, limited access to genetic counseling and testing services, and inadequate follow-up systems to support long-term patient management [1, 13].

#### Provider-level facilitators

Clinical and professional support systems facilitated the effective delivery of cancer prevention services for individuals with Lynch Syndrome [13]. National guidelines, such as those from the National Institute for Health and Care Excellence, provided clear clinical direction, improving provider confidence and helping standardize prevention recommendations [9]. High baseline knowledge among Lynch Syndrome patients supported shared decision-making, while collaboration among healthcare professionals reduced individual provider burden [9, 13]. System-level facilitators, including integrated care pathways, inter-practice agreements on evidence-based prevention strategies, and clear prevention policies, further supported consistent and comprehensive delivery of preventive care [9].

## Discussion

Clinical and professional support systems facilitated the delivery of cancer-prevention services to individuals with Lynch Syndrome [9, 13]. National guidelines, such as those from the National Institute for Health and Care Excellence (NICE), provided clear clinical direction, improving provider confidence and standardizing prevention recommendations [9]. Additionally, patients’ baseline knowledge supported shared decision-making, while professional collaboration reduced provider burden [13–16]. System-level facilitators, including integrated care pathways, inter-practice agreements on evidence-based strategies, and clear prevention policies, further promoted consistent and comprehensive preventive care [9, 17, 18].

## Strengths and limitations

This integrative review demonstrated several key strengths. A detailed search across multiple databases enhanced coverage of the literature and minimized selection bias, while clearly defined inclusion and exclusion criteria ensured a transparent and replicable study selection process. Methodological quality was systematically assessed using established Joanna Briggs Institute criteria, strengthening the rigor of the review [12]. The use of a strong theoretical framework provided a structured approach for organizing findings and linking results to both theoretical understanding and potential intervention development [10]. Additionally, the inclusion of both patient and provider perspectives enabled a more comprehensive understanding of the barriers and facilitators influencing cancer prevention in Lynch Syndrome.

Several limitations should be considered when interpreting these findings. The small number of included studies (*n* = 4) limited the strength of conclusions and the ability to examine subgroup differences. Most studies were conducted in the United States and the United Kingdom, which restricts generalizability to other healthcare systems, and the limited ethnic diversity of study samples further constrains applicability to broader populations. All included studies used qualitative designs with relatively small sample sizes, preventing the quantification of the prevalence of identified barriers and facilitators. Additionally, recruitment methods that relied on online engagement may have introduced selection bias by overrepresenting individuals with greater technological access, higher education levels, or stronger engagement with healthcare systems.

## Conclusion

Lynch Syndrome is a significantly prevalent hereditary cancer syndrome that necessitates comprehensive prevention to lower cancer risks in families. This review highlights major barriers and facilitators in prevention adherence among individuals and providers. Successful prevention requires addressing individual, systemic, and provider factors through coordinated, sustained efforts. Facilitators such as family support, trusted providers, and guidelines can boost adherence to prevention. Evidence from specific interventions, such as Patient Decision Aides, suggests potential for broader, personalized strategies that consider family circumstances and healthcare contexts. Advances in genetic testing offer new opportunities but also pose responsibilities for healthcare systems, demanding interdisciplinary collaboration to translate genetic insights into better health outcomes. Building strong research collaborations linking evidence to practice is essential for future progress. Strategies should incorporate family-centered approaches that address education about Lynch Syndrome, communication, cascade testing, and intergenerational risk management, respect personal and family values, and support decision-making.

## Appendix A

### Theoretical framework: the modified health belief model

**Figure 1:**
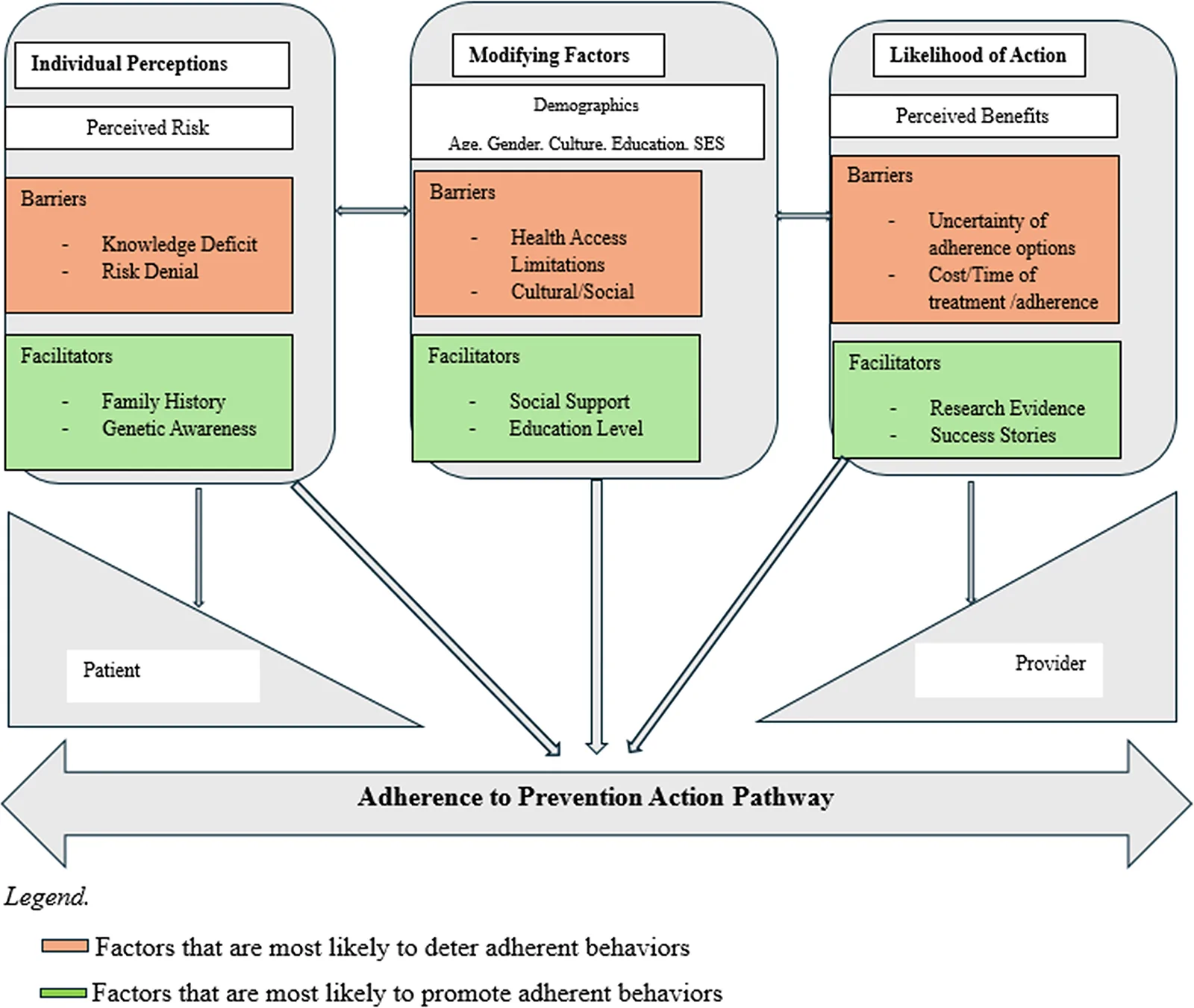

## Appendix B

### Flow Diagram for Article Selection Via Covidence

**Figure 2:**
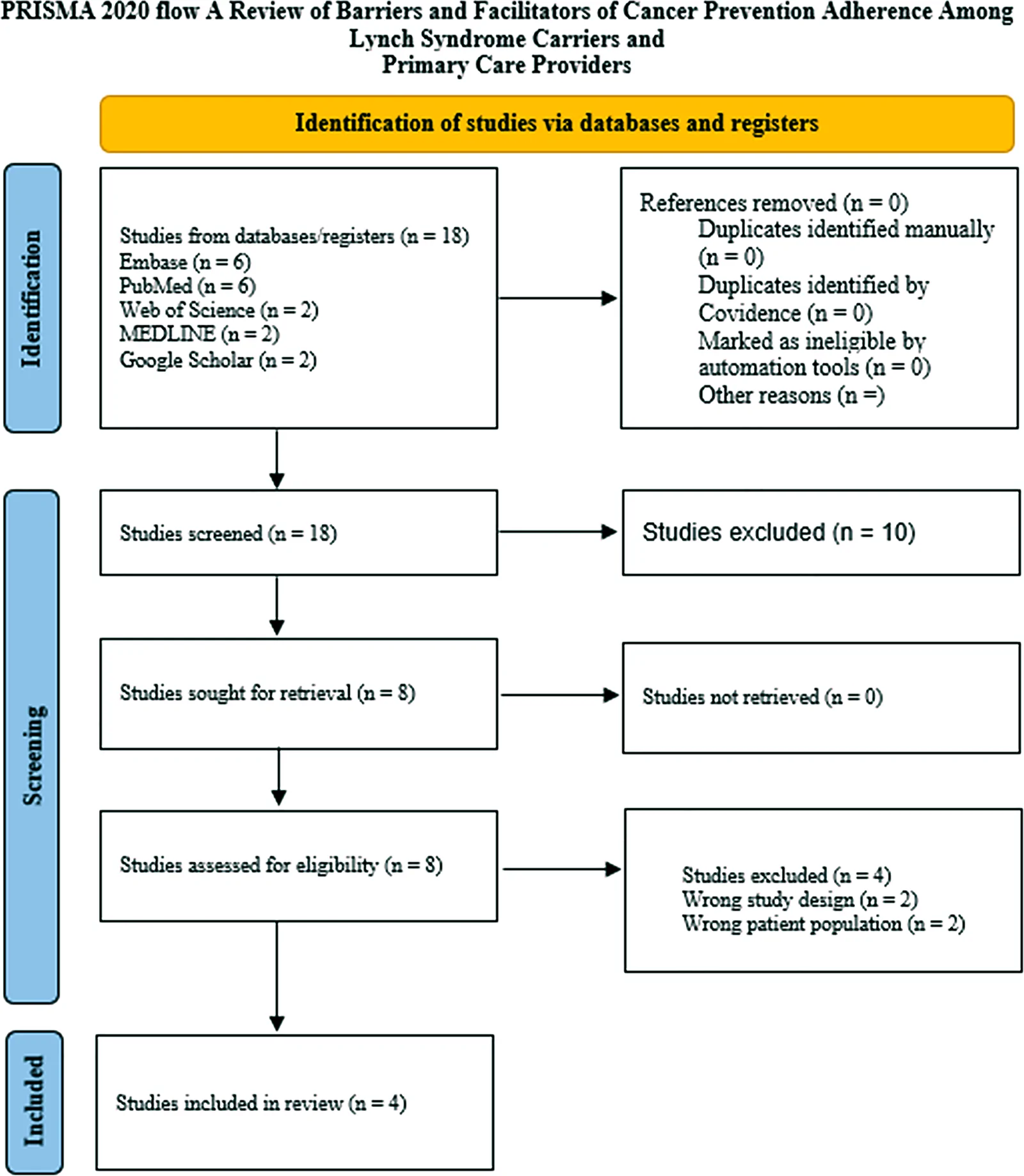

## Appendix C

### JBL quality appraisal criteria

**Figure 3:**
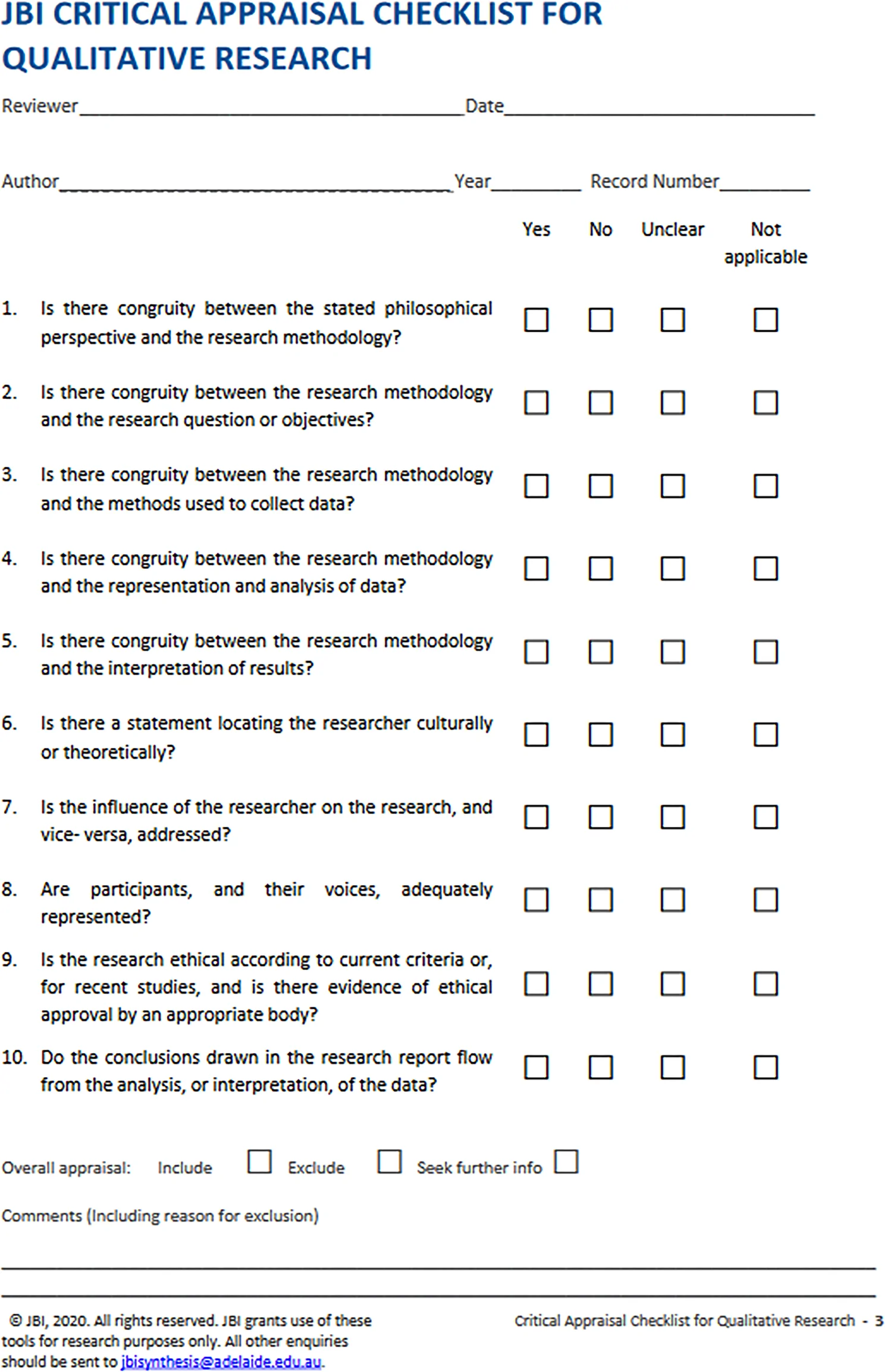

## Appendix D

### Code sheet for data extraction on the review for barriers and facilitators to cancer prevention among Lynch Syndrome carriers and primary care providers

**Table:** 

The study, First Author, and Purpose
Design: • Quantitative • Qualitative
Key Words
Variables of Interest
Theoretical Framework
Theoretical Definitions
Operational Definitions
Setting
Sample • Number of Participants • Age • Race /Ethnicity • Nationality • Education • Socioeconomic Status
Data Analysis
Outcomes Description • Barriers • Facilitators
Data Interpretation
Clinical Implications and Recommendations
Conclusion
Notes

## Data Availability

The data supporting the findings of this study are available within the article and its referenced sources.

## Abbreviations

HBM: Health Belief Model
LS: Lynch Syndrome
NICE: National Institute for Health and Care Excellence
PCP: Primary Care Provider
WCRF: World Cancer Research Fund
AICR: American Institute for Cancer Research
JBI: Joanna Briggs Institute
